# Prevalence of Anterior Segment Diseases on a Remote Island: A Telemedicine-Based Study Using the Smart Eye Camera

**DOI:** 10.7759/cureus.86759

**Published:** 2025-06-25

**Authors:** Robin Kuroiwa, Takahiro Mizukami, Hiroki Nishimura, Rohan J Khemlani, Shintaro Nakayama, Eisuke Shimizu, Shinsuke Kobayashi

**Affiliations:** 1 Ophthalmology, OUI Inc., Tokyo, JPN; 2 Ophthalmology, Fuchu Eye Center, Izumi, JPN; 3 Ophthalmology, Yokohama Keiai Eye Clinic, Yokohama, JPN; 4 Internal Medicine, Yushoukai Panauru Clinic, Kagoshima, JPN

**Keywords:** cataract, ophthalmic screening, pterygium, smart eye camera, telemedicine

## Abstract

Background and aim: Rural and remote areas often face a shortage of ophthalmic services, particularly in aging populations. This study aimed to assess the prevalence of anterior segment diseases using the Smart Eye Camera (SEC), a portable device enabling remote ophthalmic screenings.

Materials and methods: Ophthalmic screenings were conducted using the SEC to capture anterior segment videos of 158 eyes from 79 residents of Yoron Island. These videos were analyzed remotely by ophthalmologists, focusing on eyelid conditions, conjunctival abnormalities, anterior chamber depth, and lens status.

Results: The study revealed high prevalence rates of ptosis in 24 eyes (15.1%) and cataracts in 108 eyes (68.4%). Among patients over 60 years, cataracts were observed in 71 eyes (84.5%). Additionally, five eyes (3.2%) showed evidence of pterygium, all of which occurred in patients over 60 years of age.

Conclusions: Telemedicine using the SEC effectively identified age-related anterior segment diseases in this remote island population. The findings underscore the need for continued use of portable imaging devices in underserved areas to bridge gaps in eye care access.

## Introduction

The inequitable geographic distribution of physicians is a pressing issue in many countries [1-3]. Although the total number of physicians has increased in Japan, the demand-adjusted physician supply has decreased in recent years in all regions except for urban areas [4]. Furthermore, the equity of physician distribution has steadily deteriorated since 2000 [5]. While the number of ophthalmologists in Japan has increased gradually since 2000, their uneven distribution means that rural areas, particularly remote islands, often lack permanent ophthalmologists, resulting in unmet ophthalmic care needs [5,6]. Since ophthalmic diseases are more frequently observed in elderly individuals, remote islands with aging populations are disproportionately affected by this shortage of services. Research into these regions, where the proportion of elderly residents exceeds the national average, would offer valuable insights into the broader impact of Japan's inevitable population aging. Such epidemiological studies are essential for the planning and implementation of effective prevention, treatment, and follow-up strategies not only for Japan but also for developing countries facing similar challenges. Alternative surveillance strategies are needed to move beyond the routine office visit-based approach.

One such alternative is telemedicine, which utilizes technology and information exchange to deliver medical care remotely, providing services through telecommunication tools, such as smartphones, tablets, and wireless/video devices. This contrasts with conventional models of care, where the patient must visit the ophthalmologist, a practice that can pose challenges for patients residing in rural or hard-to-reach areas [7]. Several reports in the field of ophthalmology have highlighted the utility of telemedicine for patients in remote regions, including those living on islands [8,9].

The Smart Eye Camera (SEC), a novel portable device designed for ophthalmological examinations, presents a practical solution for telemedicine [10]. This innovative technology transforms a standard smartphone camera into a tool capable of performing basic assessments of the anterior segment, including evaluations of the ocular surface. The SEC has demonstrated reliability comparable to that of conventional, non-portable slit-lamp microscopes in assessing nuclear cataracts, anterior chamber depth, tear breakup time (TBUT), corneal fluorescein staining, and tear meniscus height (TMH) [10,11]. In regions lacking ophthalmologists, elderly individuals often face difficulties accessing eye care, as they must travel to areas where specialists are available. With the SEC, non-ophthalmologists can record videos, which can then be analyzed remotely by ophthalmologists, enabling eye examinations to be conducted even in remote areas, such as isolated islands. In this study, we aimed to address the current prevalence and characteristics of anterior segment diseases by conducting ophthalmic screenings using the SEC among the residents of Yoron Island.

## Materials and methods

Study population

This monocentric study was conducted on Yoron Island, Kagoshima, Japan. Yoron Island is situated at approximately 27°N latitude and 128°E longitude, with a population of 5,072 residing in an area of 20.8 km^2^. The island experiences a humid subtropical climate, and its primary industries are tourism and agriculture.

The Ethics Committee, Kohanawa Clinic Medical Corporation, Japan (IRB no. 202411161, dated November 18, 2024) approved the research protocol, ensuring adherence to the ethical principles outlined in the Declaration of Helsinki. Verbal consent was recorded from all participants. Verbal consent was obtained from all participants, who were informed of the option to opt out of the study.

Inclusion and exclusion criteria

Videos of the anterior segment were captured using a portable slit-lamp microscope Smart Eye Camera (SEC) (Tokyo, Japan: OUI Inc.) at a non-ophthalmic institution, Panaul Clinic (Kagoshima, Japan) by a medical student (R.K.). E.S. instructed R.K. on how to record anterior segment videos using the SEC and confirmed that the videos obtained were of sufficient quality for diagnostic purposes. In the present study, R.K. recorded videos using the SEC at Panaul Clinic under the supervision of the attending physician, S.K. This scheme, therefore, represents a teleophthalmology model between a primary care physician and an ophthalmologist. These videos were then evaluated by ophthalmologists at Fuchu Hospital (Osaka, Japan) and Yokohama Keiai Clinic (Kanagawa, Japan). The inclusion criteria were patients who visited Panaul Clinic between September 1, 2024, and September 9, 2024, and staff members of Panaul Clinic. The exclusion criteria were as follows: (1) patients with infectious diseases who were under quarantine within the clinic, and (2) patients who declined imaging due to a lack of understanding or cooperation.

Smart Eye Camera

**Figure 1:**
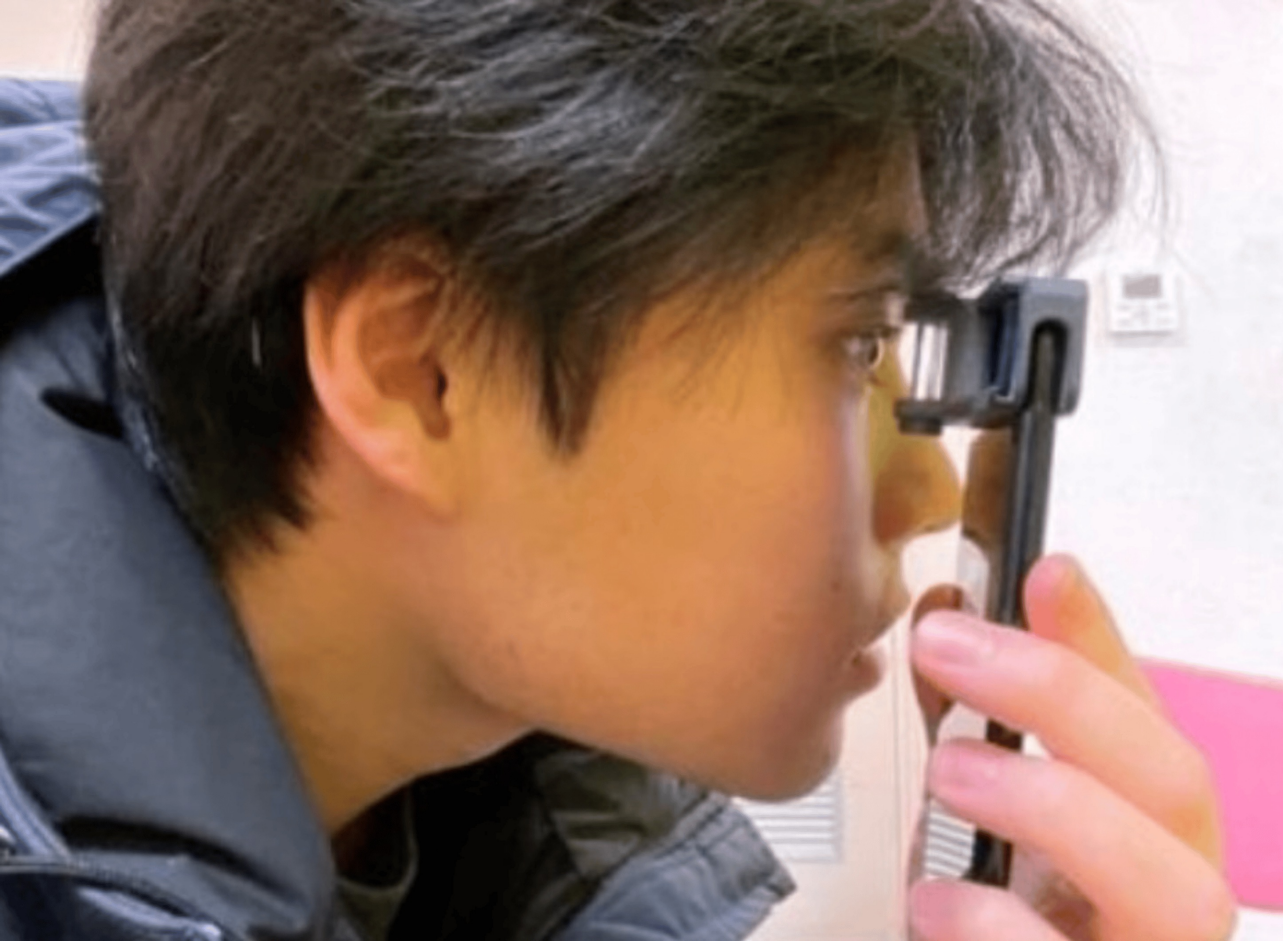
Imaging procedure and workflow. The Smart Eye Camera (SEC) modifies the iPhone's light source into a slit beam. During examination, the iPhone’s camera module (located in the upper left corner) is brought approximately 2 cm close to the patient's eye, ensuring that the slit beam illuminates the eye while recording a video. Observations were first conducted with the eyelids in their natural state, without manual assistance. When necessary, the upper eyelid was gently lifted with the thumb to assess specific parameters, such as ptosis.

The SEC is a portable device designed to function as a slit lamp. It has been approved as a medical device in Japan (registration number: 13B2X10198030101), Europe (with CE marking), Kenya, Vietnam, Cambodia, and Indonesia. The SEC is a smartphone attachment that utilizes the smartphone’s light source to create slit illumination. This enables the device to project a slit light between 0.1 mm and 0.3 mm, allowing for detailed observation of the lens without the need for mydriasis [10,12]. The actual imaging procedure is shown in Figure 1. For this study, an iPhone 7 (Cupertino, CA: Apple Inc.) was used to record videos of the anterior segment using the SEC. Video resolution was set between 720x1280 pixels and 1080x1920 pixels, with a 30 or 60 frames per second frame rate. The system is operated via a dedicated, user-friendly application that provides seamless access to the smartphone’s camera for video capture. The application also offers secure storage options, both online and offline. A teleconsultation feature is also integrated into the app, enabling remote ophthalmological evaluations. This feature facilitates virtual consultations between patients and healthcare professionals, providing a practical solution for both clinicians and patients in the field of ophthalmology [8].

Video assessments

The videos were remotely analyzed by ophthalmologists from the respective institutions. Ophthalmologists assessed the anterior segment videos recorded using the SEC via the SEC cloud-based video filing system. Observations were initially conducted in the natural state of the eyelids, without any manual assistance. When necessary, the upper eyelid was gently lifted using the thumb to facilitate the assessment of specific parameters, such as ptosis. The evaluations were performed sequentially to minimize variability, ensuring consistency across all participants. The timing of ptosis evaluation and other parameters was standardized to align with this procedure. The evaluation was divided into five parts - eyelid, conjunctiva, iris, anterior chamber, and lens - with each segment examined for specific conditions as follows: for the eyelid, the presence of ptosis, defined as a marginal reflex distance 1 (MRD-1) of less than 2 mm (i.e., the distance between the upper eyelid margin and the center of the pupil), and meibomian gland dysfunction (MGD), defined as at least one lid margin abnormality such as irregular lid margins, vascular engorgement, plugged meibomian gland orifices, or displacement of the mucocutaneous junction [13]; for the conjunctiva, the presence of hyperemia, conjunctivochalasis, and pterygium [14]; for the iris and lens, the presence of pseudoexfoliation (PE) [15]; for the anterior chamber, its depth (shallow or deep), assessed using standardized notation criteria; and for the lens, the presence of an intraocular lens (IOL) or nuclear sclerotic cataract, with cataract severity graded according to the Lens Opacities Classification System II (LOCS II) on a scale of 1-4 (with 4 being the most severe) [16].

Two ophthalmologists (T.M. and E.S.) independently evaluated all recorded videos. In cases where their assessments differed, they discussed the results together to reach a consensus. We investigated whether there was a difference in the prevalence of each condition between individuals aged 60 years and above and those under 60 years of age.

Statistical analysis

Descriptive statistics were used to describe the sample in terms of mean and standard deviation (SD). Characteristics were compared using an unpaired t-test, a chi-square test, and Fisher’s exact test. P<0.05 was considered statistically significant in all analyses. All analyses were performed using JMP Pro 17 software (Cary, NC: SAS Institute Inc.).

## Results

Patients’ characteristics

The study obtained a total of 158 eyes of 79 subjects, which is approximately 1.56% of the 5,072 inhabitants of the Island. The sample characteristics of the study were as follows: 31 males (39.2%) and 48 females (60.8%), with a mean age of 55.46±4.41, with age varying from four years old to 93 years old. Among the participants, 74 eyes from 37 subjects were from individuals aged 60 years or older, while 84 eyes from 42 subjects were from those younger than 60 years (Table 1).

**Table 1:** Prevalence of disease in patients aged under 60 years and over 60 years. ^a^P<0.05 statistically significant. ^b^Unpaired t-test. ^c^Chi-square test. ^d^Fisher’s exact test. IOL: intraocular lens; MGD: meibomian gland dysfunction; PE: pseudoexfoliation; SD: standard deviation

Variables	Under 60 years (n=74)	Age 60 years and above (n=84)	Test statistic	p-Value
Age (mean±SD) (years)	38.1±13.6	71.1±6.3	t=31.87	<0.001^a,b^
Gender (female), n (%)	48 (64.9)	48 (57.1)	χ^2^=0.138	0.7102^c^
Ptosis, n (%)	0 (0)	24 (28.6)	N/A	<0.001^a,d^
MGD, n (%)	2 (2.7)	8 (9.5)	N/A	1.000^d^
Hyperemia, n (%)	2 (2.7)	6 (7.1)	N/A	1.000^d^
Conjunctivochalasis, n (%)	0 (0)	6 (7.1)	N/A	<0.001^a,d^
Pterygium, n (%)	0 (0)	5 (6.0)	N/A	<0.001^a,d^
PE, n (%)	0 (0)	5 (6.0)	N/A	<0.001^a,d^
Anterior chamber (deep), n (%)	72 (97.3)	78 (92.9)	N/A	1.000^d^
Lens (IOL), n (%)	0 (0)	13 (15.5)	N/A	<0.001^a,d^

Eyelid

The most common eyelid condition observed was ptosis, which was present in 24 eyes (15.1%), followed by MGD in 10 eyes (6.3%). In patients under 60 years of age, no cases of ptosis were observed, and MGD was found in two eyes (2.7%). The prevalence of ptosis was significantly higher in patients aged 60 years and older; however, no significant difference was observed in the prevalence of MGD (p<0.001 and p=1.000, respectively) (Table 1).

Conjunctiva

In the conjunctiva, hyperemia was most prevalent and found in eight eyes (5.1%), followed by conjunctivochalasis in six eyes (3.8%), and pterygium in five eyes (3.2%). In patients under 60 years of age, no cases of conjunctivochalasis and pterygium were observed, while hyperemia was found in two eyes (2.7%). The prevalence of conjunctivochalasis and pterygium was significantly higher in patients aged 60 years and older; however, no significant difference was observed in the prevalence of hyperemia (p<0.001, p<0.001, and p=1.0000, respectively) (Table 1).

Presence of pseudoexfoliation 

PE was prevalent in five eyes (3.2%). In patients under 60 years of age, no cases of PE were observed. There was a significant difference in the prevalence of PE between patients aged 60 years and older and those under 60 years (p<0.001) (Table 1).

Anterior chamber depth and lens status

A shallow anterior chamber was observed in eight eyes (5.1%), while 150 eyes (94.9%) presented with a deep anterior chamber. Among the eyes evaluated, 13 (8.2%) were pseudophakic with IOLs, and no aphakic eyes were identified. The remaining 145 eyes (91.8%) were phakic. The anterior chamber of all eyes with IOLs was deep. Focusing only on phakic eyes, 72 eyes (97.3%) had a deep anterior chamber in the under-60 years age group, while 65 eyes (91.5%) had a deep anterior chamber in the over-60 years age group. Among the phakic eyes, cataract grading revealed the following distribution: 50 eyes (31.6%) were classified as grade 0, 18 eyes (11.4%) as grade 1, 66 eyes (41.8%) as grade 2, and 11 eyes (7.0%) as grade 3. Of the 145 phakic eyes, 137 (86.7%) had a deep anterior chamber, while eight eyes (5.1%) had a shallow anterior chamber (Table 1).

Representative cases

**Figure 2:**
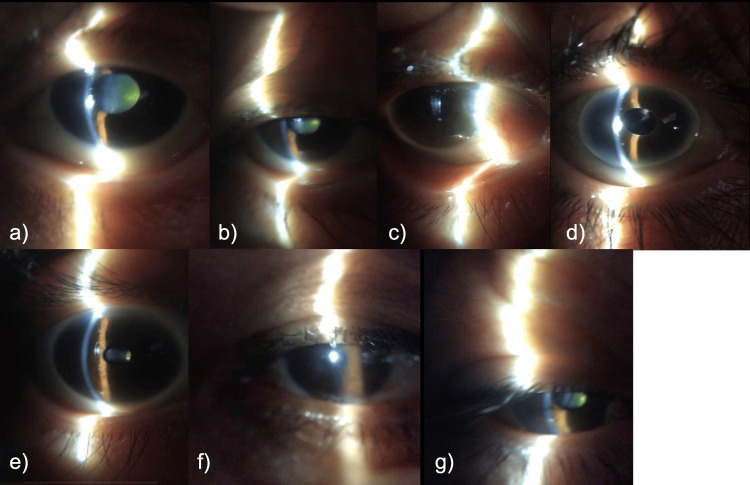
Representative cases of ophthalmologic findings in the study population. (a) A 73-year-old female with a shallow anterior chamber in the left eye. (b) An 80-year-old female with a grade 3 cataract in the right eye. Grade 3 cataract is observed, indicative of a moderate degree of lens opacity affecting visual acuity or evaluating postoperative visual outcomes. (c) A 70-year-old male presenting with pterygium in the right eye. This growth, extending from the conjunctiva onto the cornea, highlights a commonly encountered degenerative lesion in older populations. (d) A 92-year-old female exhibiting pseudoexfoliation in the right eye. Pseudoexfoliation is visualized in this case, demonstrating characteristic fibrillar material deposition in the pupillary area. (e) A 42-year-old female patient with meibomian gland dysfunction (MGD) in the right eye. Plugging of the meibomian gland orifices is observed in the lower eyelid. (f) A 93-year-old male patient with conjunctivochalasis in the left eye. (g) A 70-year-old male patient with ptosis in the right eye.

The representative images of several patients are shown in Figures 2a-2g.

## Discussion

In remote islands and rural areas where access to ophthalmologists is limited, there have been reports of non-ophthalmologists capturing videos of patients’ eye conditions, which are then diagnosed remotely by ophthalmologists to assess urgency and make diagnoses. In this study, a portable slit-lamp microscope (the SEC) was used by a medical student to capture images of the anterior segment, which an ophthalmologist subsequently evaluated through telemedicine. This study aimed to investigate the prevalence of anterior segment diseases on Yoron Island, a remote island, using the SEC for eye screenings.

This study revealed significant findings, including a low percentage of IOLs and a high prevalence of cataract grades above two among patients over 60 years of age. Specifically, 15.5% of patients over 60 years had undergone IOL implantation, in contrast to a previous study conducted on Miyako Island, located 400 km south of Yoron Island, where the prevalence was reported at 48.33% [17]. This situation is likely related to the fact that no ophthalmic surgeries are currently performed on Yoron Island. Patients requiring ophthalmic surgery must travel to the mainland or Okinawa Prefecture, which demands considerable time and resources. In this study, the prevalence of cataract grades over two in patients over 60 years of age was 82.1%. In contrast, prior research conducted on Amami Island, located 200 km north of Yoron Island, indicated a prevalence of cataract grade of only 22.3% in cohorts over 60 years of age [18]. These findings indicate a higher observed prevalence of nuclear cataracts in our study population, which may be attributed to differences in geographic, genetic, or environmental factors between the two regions [19]. Further investigation is required to determine the underlying causes of this discrepancy.

Another ultraviolet-related condition observed in this study was pterygium. The prevalence of pterygium increases with age, from 3% in the 10-20-year age group to 19.5% in those over 80 years [20]. The prevalence of pterygium is known to be high in the "pterygium belt," located between 30 degrees north and 30 degrees south of the equator, with rates ranging from 3% to 30% across different populations [21]. In this study conducted on Yoron Island, located at 27 degrees north on the periphery of the "pterygium belt," the prevalence of untreated pterygium was found to be 3.2%. Its geographic position at the edge of this region may help explain the relatively low prevalence observed. Additionally, ptosis was significantly more prevalent in individuals aged 60 years and older. Since ptosis is an age-related condition, it is natural for its prevalence to increase with advancing age. These findings suggest that the SEC effectively captures these conditions, demonstrating its utility as a diagnostic tool.

In the present study, MGD was observed in only 6.3% of participants, indicating a low prevalence. It is well known that the prevalence and severity of MGD can vary significantly depending on climate, with generally lower prevalence and milder forms observed in warm and humid regions compared to cold and dry climates [22]. Therefore, the warm environment of Yoron Island may have contributed to the low prevalence observed in this study. Moreover, ptosis was observed in 15.1% of patients in this study. Estimates of ptosis prevalence are largely based on region-specific studies, which report rates ranging from 4.7% to 13.5% in adult populations, supporting the widespread nature of the condition [23]. Therefore, the prevalence observed in this study is considered reasonable.

In remote eye screenings using the SEC, an increase in the prevalence of age-related diseases was detected, which aligns well with the findings from other studies conducted in clinical settings [19,21]. This study demonstrates the potential of telemedicine in ophthalmology to provide eye screening to remote islands and rural areas where ophthalmic services are in high demand. By utilizing devices and systems for remote consultations with ophthalmologists, effective eye screening can be delivered to populations facing geographic, financial, and resource-related limitations. In regions lacking specialists, remote interpretations of computerized tomography scans and telemedicine-based diagnoses of skin conditions are already in practice [24,25]. This study further underscores the possibility for non-specialized personnel to record eye images, which can then be evaluated by professional ophthalmologists. In areas with a shortage of ophthalmologists, it is considered beneficial for personnel, such as nurses in family medicine clinics, to capture video footage of patients' eyes using the SEC for screening purposes. This could aid in the early detection of eye diseases.

The findings provide valuable insights into the prevalence and severity of anterior segment diseases among the residents of Yoron Island. The prevalence of age-related cataracts increased with age, with the highest rates observed in individuals over 60 years of age, consistent with previous reports [26]. This study underscores the potential effectiveness of telemedicine and remote consultations for ophthalmologic care in geographically isolated regions with limited resources. This study has several limitations that should be acknowledged. First, diagnoses made using the SEC were not directly compared with those made using a conventional slit-lamp microscope. Therefore, further studies involving larger cohorts and head-to-head comparisons are needed to evaluate the diagnostic accuracy of the SEC. Second, the study did not include visual acuity or other functional outcome measures, limiting the interpretation of cataract severity and its clinical relevance. Incorporating mobile visual acuity testing in future research may provide a more comprehensive assessment of visual function. Third, although we attempted to minimize selection bias by including all patients attending a primary care clinic during the defined study period, the sample accounted for only 1.56% of the island’s population and included clinic staff. The small sample size, use of data from a single clinic, and inclusion of healthcare workers may limit the generalizability of the findings. Finally, several authors are employees, patent holders, or recipients of honoraria from OUI Inc., the manufacturer of the SEC. While image evaluations were conducted using standardized diagnostic criteria, the absence of independent observers without conflicts of interest remains a potential source of bias.

## Conclusions

This observational study suggests that ophthalmic screenings using the SEC may offer diagnostically useful information and are feasible in a primary care setting. However, due to the small and potentially biased sample, including clinic staff and only 1.56% of the island population, the findings should be interpreted with caution. No direct comparison was made with conventional slit-lamp examinations; therefore, diagnostic equivalence cannot be concluded. In regions with limited access to ophthalmologists, such as remote islands and rural communities, SEC-based screenings, performed by non-ophthalmologists, may serve as a practical tool to improve access to eye care. Future studies with larger, more representative populations and head-to-head comparisons with standard slit-lamp examinations are needed to validate these findings and assess diagnostic reliability.
